# Cytotoxicity of water supply in a Palestinian refugee camp and a Syrian informal tented settlement in Lebanon

**DOI:** 10.1371/journal.pone.0294679

**Published:** 2024-01-02

**Authors:** Michelle El Kawak, Jana Al Hassanieh, Marwa Berjawi, Mey Jurdi, Mohamad G. Abiad, Nasser Yassin, Hassan R. Dhaini

**Affiliations:** 1 Department of Environmental Health, American University of Beirut, Beirut, Lebanon; 2 Department of Nutrition and Food Sciences, American University of Beirut, Beirut, Lebanon; 3 The Laboratories for the Environment, Agriculture and Food (LEAF), American University of Beirut, Beirut, Lebanon; 4 The Ministry of Environment, Beirut, Lebanon; American University of Madaba, JORDAN

## Abstract

Deficient water, sanitation, and hygiene (WASH) significantly account for a high burden of disease across the globe. Lebanon, an Eastern Mediterranean lower-middle-income country with a polluted environment, a fragmented healthcare system, and an ongoing severe economic crisis, faces serious challenges in sustaining safe water supplies, especially in vulnerable communities, while also hosting the world highest refugee population per capita. This study aimed to examine the mutagenicity, and the estrogenic and androgenic activities of water supplies, across both a Palestinian refugee camp and a Syrian informal settlement. Water samples were collected from two targeted camps in Dbayeh and Choueifat, North and South of the Capital City Beirut, respectively, between the months of September and October 2022. Microbial and physicochemical properties of samples were determined, including fecal contamination, total dissolved solids, and various minerals and salts. Organic pollutants were extracted using pre-packed solid phase extraction (SPE) columns, and then mutagenicity of extracts was examined using the Ames test in two *Salmonella typhi* bacterial strains. The estrogenic and androgenic activities of extracts were assessed using the yeast estrogen and androgen screen tests assays (YES/YAS). Results show excessive levels of total coliforms and total dissolved solids (TDS) in samples from both sites. In addition, the water supply from the Dbayeh Palestinian refugee camp is mutagenic, while the water supply from the Choueifat Syrian informal settlement shows anti-androgen activity. Our findings provide valuable WASH baseline data in two major vulnerable communities in Lebanon, and highlight the importance of a water toxicity testing approach concomitant with a water safety plan, based on a holistic strategy that covers all stages of the water supply chain.

## Introduction

Globally, inadequate water, sanitation and hygiene (WASH) remain a public health concern, accounting for a wide panel of adverse health effects, including diarrhea, trachoma, malaria, schistosomiasis, lower respiratory infections, lymphatic filariasis, malnutrition, and malignancies [1, 2]. At the same time, monitoring for all possible toxic pollutants in drinking and domestic water is extremely difficult and expensive, despite advancement in analytical chemistry techniques. This is particularly true for hazardous chemicals that can elicit a biological effect at very low doses, such as in the case of mutagenic substances and endocrine disrupting chemicals (EDCs). Water supplies may contain unidentified contaminants and undetectable levels of toxic agents that can still cause a biological effect and pose a health risk of unknown magnitude.

More recently, there is a growing interest in examining the cytotoxic potential of water as a more comprehensive approach to water quality assessment. In a recent study, water samples collected from Lake Sevans in Armenia showed a mutagenic activity when tested in vitro [3]. Similar results were reported for drinking water obtained from six different locations in China, also demonstrating a mutagenic potential and DNA damaging activity in vitro [4]. In addition, chlorinated drinking water samples collected during the wet season in Guelma, Algeria showed an increase in chromosomal aberrations in Allium cepa root meristematic cells [5]. Furthermore, several studies reported an association between water contaminants and increased cancer risk even when detected levels are below drinking water standards [6–8]. On the other hand, EDCs are emerging as contaminants of major concern, as can act as estrogen and androgen agonists or blockers, hence impacting reproductive and developmental health in exposed populations [9, 10]. For instance, a study on drinking water from 18 sampling campaigns in an industrial area in China, showed that the majority of samples had an estrogenic activity in vitro by means of the Yeast Estrogen Screen test (YES) [11]. The same study also reported that 16% of the samples kept their estrogenic activity even after water treatment.

Lebanon, a middle-income country on the Eastern Mediterranean coast, faces major challenges in sustaining safe water supplies due to deficiencies in water quality monitoring, water treatment, and water management from source to distribution network, and ultimately to end-user taps. As a result, water supplied through distribution networks constitutes a potential health risk [12]. According to a previous World Health Organization (WHO) report on the country environmental burden of disease, more than 200 deaths are annually attributed to inadequate WASH in Lebanon [13]. On the other hand, Lebanon has been a shelter for refugees since its declaration as a sovereign state, and is currently hosts the largest number of refugees per capita in the world [14]. In addition to half a million existing Palestinians, the influx of Syrian refugees to Lebanon rapidly increased since 2011, to reach 1.5 M as a result of the Syrian conflict, according to a United Nations High Commissioner for Refugees (UNHCR) report [15]. Hosting refugees and displaced populations has further drained already limited shelter, services, and resources, hence toppling the already deteriorating infrastructure in the country, with a particular increase on water demand by 20%, added to a series of crises that included the COVID-19 pandemic, the Port of Beirut blast (August 2020), the solid wastes crisis, the recent waterborne cholera outbreak, and the country’s ongoing economic and financial crisis [16]. The pandemic resulted in ruptures in supply chains of WASH systems and interruption of service, as well as in a decline in response due to restricted movement of utility staff, hence compromising provision of safe water supply [17]. Additionally, the Port of Beirut blast severely damaged several water pumping stations and wastewater treatment plant facilities, that are part of the Water Establishment of Beirut and Mount Lebanon (EBML) network, which also supplies some refugees camps [18]. On the other hand, a recent assessment of water quality in Lebanese rivers showed that many sites are contaminated with organic material, heavy metals, and microorganisms [19]. In addition, the disintegrating water distribution network suffers from sewage infiltration due to inadequacy of wastewater collection systems, and leaking pipes [12]. In parallel, refugee camps and informal settlements rely on multiple water supply sources including public water networks, wells, and public reservoirs; reports on water quality in refugee camps are alarming [20]. For instance, drinking water in the Shatila Palestinian refugee camp in Beirut previously showed high levels of fecal coliform bacteria in water dispensers, including those placed in the camp preschools [21]. Similar patterns are seen in Palestinian refugee camps in the in the West Bank and Jordan, and in the Domiz Syrian refugee camp in Iraq, showing high levels of total dissolved solids, elevated nitrate, and presence of total coliform and E. Coli [22–24]. However, in Lebanon, data on water quality in refugee camps and informal settlements is scarce [25, 26].

This study is motivated by the unusually high influx of refugees to Lebanon, and the existing data gaps on water health risk and safety in these vulnerable communities. In particular, we aimed to examine the mutagenicity, and the estrogenic and androgenic activity of water supplies in two refugee habitats in Lebanon and to characterize water microbial and physicochemical properties.

## Materials and methods

### Site selection and assessment

The Dbayeh Palestinian refugee camp and the Choueifat El-Oumara Syrian informal tented settlement (ITS) were conveniently selected in order to capture the variation of WASH infrastructure, across different groups of refugees and their habitats. The sites were chosen in two semi-urbanized areas North and South of the capital city Beirut in order to capture variations in patterns of power outages, pollution levels, and diverse proximity to industrial and agricultural settings. Both sites do not have a managing authority, hence no official access permits were required. However, access to the Palestinian refugee camp and the Syrian ITS was facilitated through verbal communication with the United Nations Relief and Works Agency (UNRWA) and the UNHCR, respectively, which are the main UN agencies in charge of humanitarian assistance at sites.

The Dbayeh camp, with a surface area of 84,300 m^2^, is located around 12 km North of Beirut, and hosts 4,500 registered refugees [14]. On the other hand, the Syrian informal tented settlement in Choueifat El-Oumara is located around 12 km South of Beirut, with no data on its surface area or the number of residents. Camp delegates were identified onsite, and an assessment survey was conducted in order to collect the following information: number of inhabitants, primary and complementary sources of water and their usage purposes, potable water distribution system, domestic water distribution system, existence and type of water treatment scheme with quality monitoring means, and major water complaints.

### Water samples collection

The water collection process was carried out between the months of September and October 2022. At both sites, water was collected in quadruplicates from a main camp container/pool, and randomly from individual domestic water tanks. Obtained replicates from each source were then combined to construct four composite water volumes representing each site for an array of tests. Briefly, from each site, a 60L composite sample was collected in stainless steel buckets for mutagenicity testing, a 4L composite sample collected in amber glass bottles for estrogenic and androgenic activity testing, 1L sample collected in High-Density Polyethylene (HDPE) plastic bottles for water quality parameter testing, and 300 ml sample captured in sterile glass bottles with 1g of sodium thiosulfate for microbiological testing. All samples were stored at 4°C until further analysis. Microbial and physicochemical properties were initiated immediately upon arrival to the lab, while all other tests were initiated within 24–48 hours of collection time.

### Water quality parameter measurements

Samples were analyzed for their physicochemical and microbiological quality using certified prepared reagents under the US Environmental Protection Agency (EPA) standards. Tested parameters were selected based on the WHO’s standard protocol guidelines for drinking water quality analysis [13]. Samples were tested for total and fecal coliform, turbidity, electrical conductivity, total dissolved solids (TDS), pH, alkalinity, total hardness, calcium hardness, total chlorine, free chlorine, chlorides, nitrite, nitrate, ammonia nitrogen, orthophosphate, and sulfate (Table 1). Laboratory procedures followed the standard methods recommended by the American Water Works Association (AWWA), the American Public Health Association (APHA), and the Water Environment Federation (WEF) [27].

**Table 1:** List of analytical methods, equipment and reagents used for water quality analysis.

Analytical Parameter	Standard Analytical Method	Type of Analytical Equipment & Main Reagents
**Turbidity**	Nephelometric Method	HACH Turbidimeter 2100 p
**Electric conductivity**	Electrical Conductivity Method	HQ40D HACH Conductivity, pH, DO Meter
**Total Dissolved Solids**	Electrical Conductivity Method	HQ40D HACH Conductivity, pH, DO Meter
**pH**	Potentiometric method	HQ40D HACH Conductivity, pH, DO Meter
**Alkalinity**	Standard Acid Titration Method using Sulfuric Acid (0.02N)	Burette Titration apparatus and Bromcresol green-methyl red indicator
**Total Hardness**	Standard EDTA Titrimetric Method	Hardness Buffer (ammonium acetate), Erichrom Black T indicator (Manver 2 indicator powder pillow), EDTA, and Burette Titration Apparatus
**Calcium Hardness**	Standard EDTA Titrimetric Method	Murexide indicator (CalVer 2 powder pillow), Erlenmeyer flask, EDTA, and Burette Titration apparatus
**Free Chlorine**	DPD Method	DPD Free Chlorine Reagent Powder Pillows and DR 2800 HACH Spectrophotometer
**Total Chlorine**	DPD Method	DPD Total Chlorine Reagent Powder Pillows and DR 2800 HACH Spectrophotometer
**Chlorides**	Standard Mercuric Nitrate Titration Method	Acidified indicator, Diphenylcarbazone powder pillow, standard Mercuric Nitrate 0.0141N solution), Erlenmeyer flask, and Burette Titration apparatus
**Ammonia-Nitrogen**	Direct Nesslerization Method	DR 2800 HACH Spectrophotometer, Mineral Stabilizer, Nessler Reagent, and Polyvinyl Alcohol Dispersing Agent (PVA)
**Nitrite-Nitrogen**	Diazotization Method	NitriVer 3 Nitrite Reagent Powder Pillow (sulfanilic acid) and DR 2800 HACH Spectrophotometer
**Nitrate-Nitrogen**	Cadmium Reduction Method	NitraVer 5 Nitrate Reagent Powder Pillow and DR 2800 HACH Spectrophotometer
**Orthophosphate**	Ascorbic Acid Method	PhosVer 3 Phosphate Reagent Powder Pillows and DR 2800 HACH Spectrophotometer
**Sulfates**	SulfaVer 4 Turbidimetric Method	SulfaVer 4 Reagent Powder Pillows and DR 2800 HACH Spectrophotometer
**Total and Fecal Coliform**	Membrane Filtration Technique	Millipore Filtration Apparatus, M-Endo Broth, and M-FC Broth

### Quality assurance and control

A blank was run for every batch of samples during spectrophotometry analysis, and duplicate testing was conducted for all the parameters in order to ensure quality. In order to minimize cross contamination across samples, collection containers were thoroughly cleaned and oven-dried prior to field visits. In addition, before collection, containers were rinsed with water from the same collection source. All parameters were analyzed under standard conditions and using standard methods of water analysis [28–30]. All plastics used for analysis were rinsed twice with distilled water prior to usage. Measurements were conducted twice per sample for quality control, and the average concentration was reported. Calibration of the Multimode Microplate Reader (Berthold Technologies, Germany) is normally conducted every six months, while the calibration of the physicochemical analytical equipment (Table 1) is conducted before every measurement using standards or blanks, according to manufacturer’s instructions (HACH Turbidimeter 2100 p, HQ40D HACH Conductivity, pH, and DO Meter, and DR 2800 HACH Spectrophotometer–HACH Company, Colorado, United States). In addition, for the water quality parameter measurements, water samples were tested immediately upon arrival to the lab, while other tests were conducted within 24 hours of collection time.

### Extraction of organic water pollutants

Extraction of organic water pollutants was completed using a customized setup consisting of silicone tubing, a vacuum pump, and pre-packed solid phase extraction (SPE) columns. The 60L-volume and 4L-volume water samples were run through a Dionex^™^ SolEx^™^ (ThermoFisher Scientific, Massachusetts, United States) C18 Silica-Based SPE cartridges to adsorb organic pollutants as described [4, 11]. SPE columns were preconditioned with 5mL of methanol and 5mL ddH_2_0 prior to their use. Then, each sample was extracted with multiple 6mL C18 columns at an average flow rate of 15–20 mL/min. Columns were stored at -80°C overnight then dried using a vacuum freeze-dryer. Elution was then conducted with 1 mL of methanol, 1 mL of acetone, 2 mL of dichloromethane, and 2 mL of hexane per column. The composite eluent solution was dried using a nitrogen stream with a water bath at 40°C in order to speed up the evaporation process. Finally, the extracts were re-dissolved in 6 mL dimethyl sulfoxide (DMSO), and stored at -20°C for further analysis.

### Mutagenicity testing

Water mutagenicity was examined using the Ames test MPF^™^ 98/100 AQUA Microplate Format Mutagenicity Assay kit according to manufacturer’s instructions (Xenometrix, Allschwil, Switzerland). The test was carried out in triplicate. Briefly, overnight cultures of *Salmonella typhi*. strains *TA100*, *TA98*, and a negative control were prepared. Extracts were then subject to a 6-step serial dilution with a dilution factor of 0.5, and then bacterial cultures were exposed to the different diluted solutions in 24-well plates. In order to account for metabolic bioactivation, extracts were additionally tested for their ability to cause bacterial reverse mutations in the presence of a rodent liver fraction (S9). As positive controls, a solution combining 2-nitrofluorene (2-NF) and 4-nitroquinoline N-oxide (4-NQO) was prepared in DMSO for samples tested without S9, and a solution containing 2-aminoanthracene (2-AA) was prepared for samples tested with S9. Plates were then incubated at 37°C, with shaking at 250rpm for 90 min. After incubation, 2.6 ml of a pH-indicator medium was added to each of the 24 wells, and then each volume from the 24-well plates was transferred to 48 wells in a 384-well plate, placed into a sealable plastic incubation bag, and incubated at 37°C for 48 hours. The number of wells containing reverting colonies, identified colorimetrically as yellow, were counted for each dilution, and compared to the negative control and to each other in order to check for a dose-response relationship (S1 File).

### Endocrine disruption testing

Extracts from collected water samples were also assessed for estrogen and androgen agonist and antagonist activities in yeast cell lines using the XenoScreen XL YES/YAS assay kit according to manufacturer’s instructions (Xenometrix, Allschwil, Switzerland). The assays were carried out in duplicate. Briefly, YES/YAS strains (Xenometrix, Allschwil, Switzerland) cultures were prepared by suspending supplied yeast discs in growth medium in T25 ml flasks, and incubated on an orbital shaker at 31°C for 2 days. Then, 2–4μL of water organic extracts were added to the plates. To prepare positive controls, 17β-estradiol (E2), 5α-dihydrotestosterone (DHT), 4-hydroxytamoxifen (HT), and flutamide (FL) were dissolved in DMSO, and added to the corresponding labeled 96-well plates. Samples were then subject to an 8-step serial dilution with a dilution factor of 3.16. Then, a mix of yeast culture and test medium were added to all wells in order to test for agonistic activity, with E2 as positive control for the YES agonist plate, and DHT for the YAS agonist plate. In parallel, in separate 96-well plates, a mix of yeast culture, test medium, and the corresponding YES or YAS agonist positive control were added to all wells in order to test for the antagonistic activity, with HT as positive control for the YES antagonist plate, and FL for the YAS antagonist plate. The plates were then covered with gas permeable sealers, placed in a sealable plastic incubation bag with wet paper towels, to ensure a level of humidity preventing evaporation, and incubated for 18 hours at 31°C with a 100-RPM agitation. The reporter gene LacZ (encoding the enzyme β-galactosidase and estrogen or androgen responsive elements’ reaction) mixture was then prepared according to manufacturer’s instructions, and added to all the wells of a 96-well plate, then incubated overnight at 31°C with 100-RPM agitation. Agonistic activity was then evaluated colorimetrically -when the yellow substrate chlorophenol red-β-D-galactopyranoside (CPRG) converts into red- and quantified at optical density (OD) 570nm using TriStar^2^ LB 942 Multimode Microplate Reader (Berthold Technologies, Germany), while antagonistic activity was evaluated through the inhibition of any colorimetric change of the CPRG yellow substrate (S2 File).

### Statistical analysis

For the Ames test, statistical analysis was performed using the cumulative binomial distribution test in order to determine the mutagenicity of sampled water supplies in the different refugee camps. The cumulative binomial distribution test was used to determine a probable difference of 99% of a given sample dose compared to the solvent control. The binomial B-value indicates the probability of spontaneous mutations events for a given concentration. Samples were considered mutagenic if they show a dose-dependent at least ≥2-fold the solvent baseline, and a statistically significant Binomial B-value of ≥0.99 in reverting colony numbers upon exposure to tested water extracts compared to solvent control [31]. A binomial B-value ≥ 0.99 indicates that spontaneous mutagenic chances are extremely low (≤ 1%) and accordingly the test result would be considered positive. In cases of B-value ≤ 0.01, data points were considered significantly smaller than the mean number of spontaneous revertants, and were labeled negative for mutagenicity.

For endocrine disruption agonistic and antagonistic activity, the induction ratios (IR) were based on the obtained optical density (OD) readings for absorbance measurements of the assay plates. The induction ratios were determined after calculating a multitude of variables including the growth factor, β-galactosidase activity (relative units), limit of quantification and limit of detection according to manufacturer’s instructions. A sample dilution was considered to have an agonistic endocrine activity in cases where the Induction Ratio IR ≥ 10 in either the YES or YAS Agonist assay. In addition, a sample dilution was considered to have antagonistic endocrine activity if the Induction ratio IR ≤ 50 in either the YES or YAS Antagonist assay [32].

## Results

### Assessment survey results

The Dbayeh Palestinian Refugee camp is located near a highly populated residential area, and surrounded by agricultural activities, potentially exposed to anthropogenic sources of pollution. Around 540 Palestinian families reside in the camp, with an average of seven members per family, in addition to 60 Syrian families, with an average of five members per family. The main source of domestic water is a closed concrete tank that receives water from the Water Establishment of Beirut and Mount Lebanon (EBML) distribution network. A central water supply system exists since the establishment of the camp; however, the distribution network within the camp is old and without maintenance. Many residents have their own domestic water tanks, which are directly supplied from the main concrete tank. Households with no access to the distribution network fill their domestic water tanks through self-paid water trucks. Residents use the water from their domestic water tanks for cooking, washing vegetables, personal hygiene, and other domestic purposes, while occasionally chlorinating the water storage tanks. Due to power shortages, water distribution and pumping to tanks are frequently disrupted. For drinking, camp residents rely on other diverse sources of water.

The Choueifat El-Oumara Informal Tented Settlements site started to be occupied by Syrian refugees before the Syrian conflict around fifteen years ago. There are currently 50 families residing on the site, each composed of 6–10 family members. The water body in the settlement is a groundwater well pumped to a partially exposed concrete pool. The lack of a central water supply system forces residents to pump water from the pool into their domestic storage tanks. Many residents also acquire their drinking water in the form of treated water from a commercial water supplier based in their area, using UN agencies-provided allowance. Procured water is directly filled into individual tanks every 2–4 days, depending on the season and the needs. Accordingly, some tents have individual 300L volume potable water tanks and 10,000L volume domestic water tanks, while others share a common 10,000L non-potable water tank. Residents’ major complaints about the water quality include power shortage, presence of rodents and fungus around and inside the water tanks, and prevalent skin allergies among infants. No water treatment is employed prior to distribution. The exposed concrete pool is also exposed to anthropogenic sources of pollution such as residential activities, wastewater intrusion, agricultural runoffs, and leachate from nearby open dumps.

### Water quality results

#### Physical and microbiological quality

Results show excessive total coliforms and total absence of fecal coliform in both the Syrian informal tented settlement water supply in Choueifat and the Palestinian refugee camp water supply in Dbayeh. In addition, turbidity was relatively high in all collected samples. High levels of TDS are evident particularly in the Choueifat water supply, and can be explained by the high level of pollution. Conductivity was also detected in all collected domestic water samples from both camps (Table 2).

**Table 2:** Mean values of the physical and microbiological parameters of the water supply in the Dbayeh Palestinian refugee camp and the Syrian informal tented settlement of Choueifat.

	Physical Parameters			Microbiological Parameters	
	Turbidity (NTU)	Conductivity (μs/cm)	TDS (mg/L)	Fecal coliform (CFU/ 100ml)	Total coliform (CFU/ 100ml)
**Choueifat Syrian Informal Settlements**					
**Pool**	0.78^ǂ	2650*^ǂ	1330*^ǂ	0	too numerous to count*^ǂ
**Composite Water Tanks (1)**	0.59^ǂ	2730*^ǂ	1370*^ǂ	0	too numerous to count*^ǂ
**Composite Water Tanks (2)**	0.61^ǂ	2700*^ǂ	1360*^ǂ		too numerous to count*^ǂ
**Drinking Water Tanks**	0.53^ǂ	290	150	0	too numerous to count*^ǂ
**Dbayeh Palestinian Refugee Camp**					
**Composite Water Tanks**	0.88^ǂ	700*ǂ	365	0	too numerous to count*^ǂ
**Main Storage Tank**	0.56^ǂ	800*ǂ	480	0	too numerous to count*^ǂ

* exceed the Maximum Admissible Levels/Concentrations of the LIBNOR national standard for drinking water (2016)

^exceed the Maximum Admissible Levels/Concentrations of the WHO Guidelines Drinking water quality guidelines Standards (2022b)

^ǂ^ exceed the Maximum Admissible Levels/Concentrations of the US EPA Primary Drinking Water Regulations (2022c)

#### Chemical quality

The water was alkaline in all collected samples. Hardness, which includes carbonate and non-carbonate, was also found to be high in all collected domestic water samples from both camps. As for sulfates and orthophosphate levels, all tested samples were below the maximum admissible concentrations. Similarly, Ammonia-Nitrogen, Nitrate-Nitrogen, and Nitrite-Nitrogen were below acceptable levels set by the Lebanese national Standards Institution (LIBNOR) in almost all samples [33]. Chloride levels were excessive in the pool and composite water tank samples, while total chlorine and free chlorine levels were relatively low in all tested samples (Table 3).

**Table 3:** Mean values of the chemical parameters of the water supply in the Dbayeh Palestinian refugee camp study area and the Syrian informal tented settlement of Choueifat.

	Chemical Parameters											
	pH	Alkalinity (mg/L as CaCO_3_)	Total Hardness (mg/L as CaCO_3_)	Calcium Hardness (mg/L as CaCO_3_)	Total Chlorine (mg/L as Cl_2_)	Free Chlorine (mg/L as Cl_2_)	Chlorides (mg/L as Cl-)	Nitrite Nitrogen (mg/L as NO_2_^-^-N)	Nitrate Nitrogen (mg/L as NO_3_^-^-N)	Ammonia Nitrogen (mg/L as NH_3_-N)	Orthophosphate (mg/L as PO_4_^3-^)	Sulfate (mg/L as SO_4_^2-^)
**Choueifat Syrian Informal Settlements**												
**Pool**	7.8	280	775*^	585*^	0.05	0.02	613*^ǂ	0.013	11.15*ǂ	0.275	0.38	117
**Composite Water Tanks (1)**	7.8	272	700*^	565*^	0.03	0.02	625*^ǂ	0.009	9.2	0.29	0.59	121
**Composite Water Tanks (2)**	7.9	290	725*^	625*^	0.06	0.04	600*^ǂ	0.007	9.4	0.29	1.06	130
**Drinking Water Tanks**	8.1	40	100	60	0.1	0.05	55	0.009	1.7	0.23	0.16	12
**Dbayeh Palestinian Refugee Camp**												
**Composite Water Tanks**	7.9	245	300	250	0.035	0.03	70	0.011	4.1	0.23	0.32	40
**Main Storage Tank**	7.8	280	310	265	0.045	0.04	75	0.007	4.45	0.26	0.36	39

* exceed the Maximum Admissible Levels/Concentrations of the Libnor national standard for drinking water (2016)

^exceed the Maximum Admissible Levels/Concentrations of the WHO Guidelines Drinking water quality guidelines Standards (2022b)

^ǂ^ exceed the Maximum Admissible Levels/Concentrations of the US EPA Primary Drinking Water Regulations (2022c)

### Mutagenicity & endocrine disruption test results

The Ames test results showed that the composed water sample obtained from the Dbayeh Palestinian refugee camp was mutagenic with *TA98 Salmonella typhi*. bacterial strains in the presence of rodent liver extract (S9) (2.47-fold over baseline, with a B-Value > 0.99). All other tested samples were statistically negative.

On the other hand, the YES/YAS assay revealed an androgen (YAS) inhibitory antagonistic activity in the Choueifat water samples across all dilutions, showing an induction ratio IR ≤ 50 (IR_avg_ = 16.73). All other tested samples and results were negative.

## Discussion

Findings from this study indicate pollution in refugee camps water supply from multiple sources. At the same time, the quality of water may also be affected by the deterioration of distribution networks. In addition, intermittent distribution of piped water supplies may lead to pressure imbalance and inflow of foreign matter. Our results show excessive total coliforms in both Syrian informal tented settlement and Palestinian refugee camp water supplies in Choueifat and Dbayeh, respectively. These results are in accordance with previous studies conducted in collaboration between the National Center for Scientific Research (LNCSR) and a number of academic institutions between 2014 and 2020, reflecting on high levels of coliforms in piped water supplies for Greater Beirut and Mount Lebanon areas [34]. Additionally, our results are also consistent with a recent nationwide assessment of water quality in fourteen rivers in Lebanon, revealing high levels of both coliform and E. coli [35, 36]. In the absence of fecal coliform, surface water effluents and surrounding small-scale agricultural activities in the neighborhood of camps, are the main sources that can explain the observed results.

In addition, turbidity was relatively high in all collected samples, which may be due to the highly porous nature of limestone, or to the deterioration the distribution network, or most probably, in this case, to the openly exposed collection tanks and household storage tanks, particularly in the Dbayeh Camp. Turbidity may also be a reflection of the suspended solid matter that comes from domestic, agricultural, and industrial activities. High levels of TDS were evident in the Choueifat informal tented settlement water supply, due to presence of increased levels of salts such as calcium, magnesium, potassium, sodium, bicarbonates, chlorides and sulfates, and organic matter, most probably due to seawater infiltration, particularly that the samples were collected at the peak of the dry season in coastal locations [37]. Further research is required in order to identify the sources of pollution. At the same time, the rise in ambient temperatures in air combined with increased water withdrawals may also elevate total dissolved solids and explain the observed results [38]. These rises in individual ions (such as sodium or chloride) and in TDS threaten water supplies, agricultural activities, and the human health [39]. In addition, alkalinity was high, supporting the almost-alkaline pH, which may be explained by the natural formation of groundwater wells and the buffering effects, which turn water from a weak acid into an alkali, through a Ca and Mg reaction with carbon dioxide (CO_2_) forming carbonates and bicarbonates. The limestone nature of the aquifers may also cause a higher deposition of minerals in the water [40]. Alkalinity can also result from agricultural runoff carrying phosphate and potassium, and wastewater contamination. Hardness, which includes carbonate and non-carbonate, was also found to be high in all collected water samples from both camps. Non-carbonate hardness is attributed to chlorides and sulfates which could result from seawater infiltration, and to a lesser extent from organics present in the nearby industrial effluent and agricultural runoff. Seawater intrusion is common in coastal areas in Lebanon [41]. The intrusion levels are further aggravated by the over withdrawal of groundwater over the last decade [42]. Chlorides may also result from sewage contamination, which can also explain the observed nitrogen levels. However, the absence of fecal coliform in our sample precludes us from making such conclusions. Elevated TDS can cause gastrointestinal irritation, while water hardness can affect the skin’s pH balance, weakening it as a barrier against harmful bacteria and infections [43], and may cause calcification of household plumbing systems [44].

On the other hand, our findings revealed mutagenicity of the water from the Dbayeh Palestinian refugee camp. Mutagens are agents that damage genetic material, potentially resulting in genetic abnormalities and disorders, and may lead to tumor formation. Mutagenic activity of water supply is due to contamination by natural or man-made pollutants such as nitrates, polycyclic aromatic hydrocarbons (PAHs), heavy metals, chlorination disinfection by products, pesticides, and others [45]. The positive mutagenic results in *TA98* Salmonella strains with post-mitochondrial rodent liver fraction (S9) indicate that water samples from the Palestinian camp have the potential of causing frameshift mutations following metabolic bioactivation. Frameshift mutations are produced by either the addition or deletion of DNA bases creating altered DNA synthesis and mutated DNA sequences, and usually result in radical changes in targeted protein sequences [46, 47]. These findings are alarming and should be followed upon, especially that the camp acquires its tap water from the Establishment of the Water of Beirut and Mount Lebanon (EBML), which supplies large residential and commercial districts in the capital city Beirut and Mount-Lebanon. These findings are similar to those from a Chinese study on river water, where results also revealed potential frameshift mutagenicity of collected samples, and effects were linked to the presence of aromatic amines and/or polycyclic hydrocarbons in the water body, mainly from industrial runoff [48]. Given both the Dbayeh proximity to industrial and agricultural activities, the found mutagenic activity of water supply is thus justified. The identity of the mutagens still needs to be determined as with the Chinese study. Another more recent study on Hrazdan river water in Armenia, which receives effluents from agricultural and industrial enterprises, revealed mutations in stamen hair, indicating the potential mutagenic and clastogenic potential of contaminated water [49]. This further iterates the water mutagenic etiology from industrial and agricultural activities. In addition, a study investigating tap water quality in Sardinia, Italy detected chemicals capable of inducing frameshift mutations, as they induced point mutations in the *TA98* strain Salmonella bacteria, while results in the *TA100* strain were borderline and only observed at the highest tested dose [50]. Comparable to the Dbayeh camp conditions, the tested water, collected from artificial basins in Sardinia runs through an exposed poorly maintained distribution network [50]. Similarly, an Ames test on suspended particulate matter from the Seine River estuary showed mutagenicity in *TA98* strains with S9 mix, confirming frameshift mutagens in water samples collected downstream of the outlet of an industrial wastewater-treatment plant [51]. Similar correlations with industrial discharges were found for water samples obtained from a river in Brazil [52]. In summary, our findings of potential water mutagenicity may be explained by industrial and agricultural activities upstream of the camp water supplies.

Furthermore, our results showed a positive endocrine disruption activity, particularly an anti-androgen activity for extracts from the Syrian ITS water supply. Environmental endocrine-disrupting chemicals (EDCs) are agents in water, air, soil, or food sources that could interfere with the normal function of the body’s endocrine system, and subsequently impair the development and fertility in exposed subjects. The source of EDCs contamination in water may vary, originating from water disinfection processes, or release from industry, agricultural and livestock activity, or sewage contamination. EDCs in water may include chlorination disinfection byproducts, fluorinated compounds, bisphenols, phthalates, pesticides, and natural or synthetic estrogens. Ligands that exhibit anti-androgen effects can bind to androgen receptors, and consequently interrupting signal transduction, and potentially leading to an increased cancer risk [53]. According to the literature, exposure to EDCs can play a major role in infertility, in addition to an increased risk of testicular cancer and cryptorchidism in male embryos, and increased breast cancer risk [54]. In particular, androgen antagonists were associated with abnormal development of the genital tract in male fetuses in utero [54], and atrophic ventral prostate and seminal vesicles in adult male rats [55]. Our findings are in accordance with a US study investigating bioactivity in public drinking water utilities in Iowa [56]. More than 50% of the samples showed androgenic activity, similarly to the water samples collected from the Choueifat settlement which is also in proximity to agricultural areas [56]. In summary, our findings indicate that a specific endocrine disrupting activity is found in water supplies in one of many informal tented settlement scattered along the Lebanese territories, and suggest potential avenues for future research to characterize EDCs exposures for epidemiological studies, and identify predictors of EDC activity [57].

While our study strength lies in targeting refugees and displaced populations water supplies using validated in vitro models, it may be limited by several factors. First, sampling was confined to two refugee camps that were conveniently selected and inspected, and hence results are not considered representative of the entire refugee population in Lebanon. A wider-scale study with a much larger budget should be conducted in the future to confirm observed results. Second, sampling was conducted at the end of the dry season, hence findings do not reflect seasonal variations, particularly dilutions during the wet season. In addition, in vitro studies may not always reflect actual effects in intact human organisms, hence limitations in extrapolation may not be ruled out.

## Conclusion

Mutagenic and anti-androgenic activity were found in water supply from two targeted refugee camps, while microbial and physicochemical parameters exceeded the maximum admissible concentrations, hence the need to take more measures to ensure water safety. Although our results may not by themselves be enough to make inference into the general refugee population in Lebanon, however, they are the first of their kind in the region and are indicative of the water quality and water mismanagement particularly in vulnerable groups and surrounding disadvantaged communities. The recent cholera outbreak that started in a Syrian refugee camp in Lebanon is a striking example of how severe a weak water management infrastructure can be. The outbreak demonstrated that the impact of poor access to safe water and poor WASH for refugees goes beyond the boundaries of the camps themselves to reach the entire hosting nation and that deterioration in water quality is becoming an observed pattern in informal urban and semi-urban areas and settlements in the country. In particular, the anti-androgenic and mutagenic activity in water supply, partly supplied from the water establishment, are alarming and indicate that sustainable solutions for the water crisis in Lebanon are inevitable and should address water safety and optimize water treatment processes. This study highlights the importance of introducing a regular monitoring scheme for the biological activity of tap water, in order to better assess its health impact. National testing of water mutagenicity and endocrine disrupting activity can help advance assessment of WASH burden of disease, forecast potential epidemic outbreaks, and serve as prevention.

At the same time, our findings highlight the need for durable solutions for refugees, and may be useful to inform decision-making at the level of international donors and local authorities, particularly in adopting a more comprehensive all-inclusive sustainable refugee management framework, taking into account WASH standards, while arranging for all refugees and displaced groups to safely return to their countries of origin. As a mitigation strategy, we recommend developing a water safety plan aiming to profile the sources and to document and manage sources of pollution on a national scale. The anticipated plan should be developed and implemented by the Regional Water Establishments (RWEs), covering all stages of water supply from source to end-user, according to the WHO guidelines, and would include identification of all possible pollution sources and their characteristics, as well as the eventual efficient improvement of the water supply system assessment, process management, and operational monitoring, especially in vulnerable communities like refugees, displaced individuals, and people living below the poverty line.

## Supporting information

S1 File Ames test number of positive colonies dataset. The number of wells containing reverting colonies, identified colorimetrically as yellow, counted for each dilution. This raw data is used to calculate the mean number of positive wells, the standard deviation, and the fold increase over the baseline and the Binomial B-value, using Excel. The resulting calculations determine the mutagenicity of the samples at specified concentrations.
(XLSX)

S2 File YES/YAS assays OD readings dataset. YES and YAS agonistic and antagonistic activity was evaluated colorimetrically by reading the optical density (OD) at 570nm and 690nm of the assay plates using TriStar^2^ LB 942 Multimode Microplate Reader (Berthold Technologies, Germany). This raw data is used to calculate the Growth factor, β-galactosidase activity, and the induction ratio, using Excel. The resulting calculations determine the estrogen and androgen agonist and antagonist activities of the samples.
(XLSX)
